# Hypoxia primes human normal prostate epithelial cells and cancer cell lines for the NLRP3 and AIM2 inflammasome activation

**DOI:** 10.18632/oncotarget.8594

**Published:** 2016-04-05

**Authors:** Ravichandran Panchanathan, Hongzhu Liu, Divaker Choubey

**Affiliations:** ^1^ Cincinnati VA Medical Center, Cincinnati, OH 45220, USA; ^2^ Department of Environmental Health, University of Cincinnati, Cincinnati, OH 45267, USA

**Keywords:** hypoxia, prostate, inflammasome, inflammation, cancer

## Abstract

The molecular mechanisms by which hypoxia contributes to prostatic chronic inflammation (PCI) remain largely unknown. Because hypoxia stimulates the transcriptional activity of NF-κB, which “primes” cells for inflammasome activation by inducing the expression of NLRP3 or AIM2 receptor and pro-IL-1β, we investigated whether hypoxia could activate the NLRP3 and AIM2 inflammasome in human normal prostate epithelial cells (PrECs) and cancer cell lines. Here we report that hypoxia (1% O_2_) treatment of PrECs, prostate cell lines, and a macrophage cell line (THP-1) increased the levels of NLRP3, AIM2, and pro-IL-1β. Further, hypoxia in cells potentiated activation of the NLRP3 and AIM2 inflammasome activity. Notably, hypoxia “primed” cells for NLRP3 and AIM2 inflammasome activation through stimulation of the NF-κB activity. Our observations support the idea that hypoxia in human prostatic tumors contributes to PCI, in part, by priming cells for the activation of NLRP3 and AIM2 inflammasome.

## INTRODUCTION

The availability of optimum levels of oxygen to cells and tissues *in vivo* is critical for normal cellular homeostasis [1, 2]. The availability of sub-optimal levels of oxygen (or lack of it) to cells due to an infection-associated inflammation, injury, or noxious agents contributes to cell death and chronic inflammation in a variety of human diseases, including cancers [3–8]. However, the molecular mechanisms through which hypoxia in solid tumors and tumor cells contributes to the development of chronic inflammation remain largely unknown.

The oxygen-responsive hypoxia-inducible factor (HIF), which consists of an unstable α subunit and a stable β subunit, plays an important role in adaptation to hypoxia through transcriptional regulation of a set of genes that encode for survival proteins [1, 2]. Further, the expression of HIF-1α is transcriptionally up-regulated by NF-κB transcription factor [9–11]. In the presence of oxygen, members of the conserved Egl-Nine (EGLN) gene family (such as EGLN1, EGLN2 and EGLN3) that encode for prolyl hydroxylases in most cell types hydroxylate the HIFα subunit [1, 2]. The hydroxylated HIFα in cells is polyubiquitinated and degraded. Under low-oxygen conditions (*e.g*, at 1% O_2_), HIF-1α is stabilized and it stimulates the transcription of a set of target genes [12, 13] and activates the transcriptional activity of NF-κB [14–17], a master regulator of genes that encode for proinflammatory cytokines such as IL-1β and IL-18 [14, 17].

Dysregulated activation of the NF-κB transcriptional activity contributes to development of inflammation-associated prostatic diseases such as benign prostate hyperplasia (BPH) and prostate cancer [18–21]. The NF-κB family includes RelA (p65) and NF-κB1 (p105/p50) [22]. Further, the p50/RelA heterodimer is held inactive in the cytoplasm by specific binding by a member of the IκB-family of inhibitory proteins, IκBα, a transcriptional target of NF-κB. Activation of NF-κB by canonical or non-canonical pathway in hypoxic cells is critical in the transcriptional response to hypoxia that results in the expression of genes that encode for the proinflammatory cytokines [14, 17, 22].

Sterile inflammatory insults due to cyclic or chronic hypoxic conditions within solid tumors initiate an influx of myeloid cells (*e.g*., monocytes and macrophages) [8]. Myeloid and epithelial cells express cytosolic DNA sensors, such as members of the AIM2-like receptor (ALRs) and nucleotide binding and oligomerization domain (NOD)-like receptor (NLRs) family [23–26]. Members of the NLR (*e.g*., NLRP3) and ALR (*e.g*., murine Aim2 and human AIM2) family receptors form a cytosolic protein complex termed the inflammasome [23, 24, 26]. The inflammasome comprises a receptor from either the NLR or ALR-family, an adaptor protein apoptosis-associated speck-like protein containing a caspase recruitment domain (ASC), and procaspase-1 [23, 26]. Activation of an inflammasome proteolytically cleaves the pro-IL-1β (p31) and pro-IL-18 (p24) to the mature IL-1β (p17) and IL-18 (p18) respectively. Increased production of proinflammatory cytokines (*e.g*., IL-1β and IL-18) contributes to inflammation [23–26].

In most cell types, the NLRP3 inflammasome is activated by a two-step mechanism, referred to as “priming” and “activation” [25, 27]. After priming by NF-κB activating signal (such as IL-1β), which induces the expression of limiting proteins (such as NLRP3 receptor and pro-IL-1β) for the activation of NLRP3 inflammasome, the NLRP3 inflammasome is activated in a second step by damage-associated molecular patterns (DAMPs) such as ATP. Although it remains unclear how NLRP3 inflammasome responds to these very diverse stimuli, it has been proposed that the NLRP3 inflammasome is activated by ligand-induced intermediates such as reactive oxygen species (ROS), K^+^ efflux, and the lysosome destabilization [28]. The Aim2/AIM2 inflammasome is activated by self or pathogen-derived cytosolic DNA (a “danger” signal) in “primed” myeloid and epithelial cells [26, 29].

Expression of AIM2 receptor, ASC and procaspase-1 is detectable in human prostate epithelial cells (PrECs) [29], keratinocytes [30], and neuronal [31] cells. Further, the IFN-treatment of human normal PrECs increased the expression of AIM2 receptor, procaspase-1, and pro-IL-1β (p31) proteins, thus suggesting “priming” of cells for activation of the AIM2 inflammasome [29]. Notably, sensing of the cytosolic DNA (synthetic DNA poly [dA:dT]), by “primed” PrECs and prostate cancer cell line PC-3 also activated the AIM2 inflammasome activity [29].

Because hypoxia in prostatic tumors is associated with chronic inflammation and a poor outcome for prostate cancer patients [19, 21, 32, 33], we investigated whether hypoxia in human PrECs, prostate cancer and myeloid cell lines promotes NLRP3 and AIM2 inflammasome activation. We report that hypoxia “primed” NLRP3 and AIM2 inflammasome through up-regulation of the NLRP3 and AIM2 receptors, and pro-IL-1β (p31). Further, hypoxia potentiated activation of the NLRP3 and AIM2 inflammasome in prostate epithelial, prostate cancer, and THP-1 cell lines.

## RESULTS

### Hypoxia primes human normal PrECs for activation of the NLRP3 and AIM2 inflammasome

**Figure 1:**
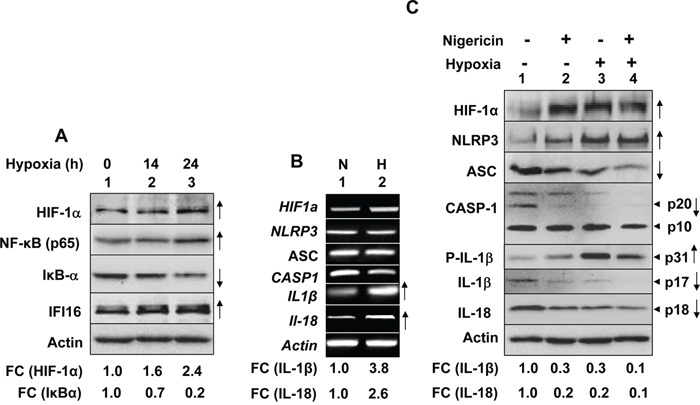
Hypoxia primed and potentiated NLRP3 inflammasome activation in human normal PrECs **A.** Sub-confluent cultures of young proliferating (passage 3) PrECs were either incubated at normoxic (O_2_ levels 20.9%) or hypoxic (O_2_ levels 1.0%) chamber for the indicated time. After the incubation, total cell lysates containing equal amounts of proteins were subjected to immunoblotting using antibodies specific to the indicated proteins. An appreciable increase or decrease in levels of a protein in hypoxia-treated cells in two or more experiments is marked by an upward or downward arrow in the right side of the Figure Further, fold changes (FC) in the levels of the indicated proteins in hypoxia-treated PrECs were calculated as described in Methods. This ratio between the protein band signal and the actin protein band signal in control cells was indicated as 1. **B.** Sub-confluent cultures of young proliferating (passage 3) PrECs were either incubated at normoxic (O_2_ levels 20.9%) or hypoxic (O_2_ levels 1.0%) chamber for 24 h. Total RNA was prepared from cells and was analyzed by semi-quantitative RT-PCR using a pair of primers that were specific to the indicated genes. Appreciable increases in levels of mRNA in hypoxia treated cells in two or more experiments are marked by upward arrows in the right side of the Figure **C.** PrECs as in the panel A, were either incubated under normoxic (lanes 1 and 2) or hypoxic (lanes 3 and 4) conditions for 14 h. After the incubation, cells were either treated with vehicle (lanes 1 and 3) or nigericin (10 μg/ml; lanes 2 and 4) for 45 min. Total cell lysates were prepared from control and treated cells and lysates containing equal amounts of proteins were analyzed by immunoblotting using antibodies specific to the indicated proteins. An arrow head in the right of figure indicates the protein band of interest, which is followed by an estimated molecular weight of the protein band. An appreciable increase or decrease in levels of a protein in hypoxia-treated cells (in two or more experiments) is marked by an upward or downward arrow, respectively. Fold changes (FC) in the levels of the indicated proteins in hypoxia-treated PrECs were calculated as described in Methods. This ratio between the protein band signal and the actin protein band signal in control cells was indicated as 1.

Because hypoxia-induced activation of NF-κB activity in cells results in transcriptional activation of genes that encode for the proinflammatory cytokines (such as IL-1β) [14, 17, 22] and epithelial cells are exposed to chronic or cycling hypoxia in prostate tumors [19, 21], we investigated whether hypoxia could activate the NF-κB activity and “prime” PrECs for activation of the NLRP3 or AIM2 inflammasome activity. As shown in Figure 1A, hypoxia treatment of proliferating (young cells; passage 2) PrECs for 0 to 24 h, which measurably increased levels of HIF-1α (compare lane 3 with lane 2 or 1), also increased levels of NF-κB (p65). Accordingly, levels of IκBα protein decreased whereas levels of the NF-κB-inducible IFI16 protein increased (compare lane 3 with lane 2 or 1). These observations suggested an activation of the NF-κB activity in human normal PrECs under our hypoxia conditions. Further, hypoxia treatment of cells appreciably increased levels of pro-IL-1β (∼4-fold) and pro-IL-18 (∼2.5-fold) mRNAs (Figure 1B), both transcriptional targets of NF-κB, but not of HIF-1α, NLRP3, ASC, and Caspase-1 mRNAs. Interestingly, the treatment, which stabilized HIF-1α protein in PrECs, appreciably increased levels of NLRP3 protein (Figure 1C, compare lane 3 with lane 1), suggesting a post-transcriptional stabilization of the NLRP3 receptor by hypoxia in human normal PrECs. Further, the treatment in cells decreased cellular levels of cleaved caspase 1 (p20), increased levels of pro-IL-1β (p31), decreased cellular levels of cleaved IL-1β (p17), and IL-18 proteins (compare lane 3 with 1), thus suggesting that hypoxia treatment of human PrECs spontaneously activated an inflammasome activity. Of interest, treatment of hypoxic cells with nigericin, an activator of the NLRP3 inflammasome [34], reduced cellular levels of cleaved caspase 1 (p20), pro-IL-1β (p31), IL-1β (p17), and IL-18 (compare lane 4 with 2) as compared with normoxic and nigericin-treated cells (Figure 1C), thus suggesting that hypoxia treatment of cells also potentiated nigericin-induced NLRP3 inflammasome activation. Similarly, hypoxia treatment potentiated the synthetic DNA (poly dA:dT)-mediated activation of the AIM2 inflammasome activity in PrECs (data not shown). Together, these observations indicated that hypoxia treatment in human normal PrECs primed cells for activation of the NLRP3 and AIM2 inflammasome.

### Hypoxia primes and activates the NLRP3 inflammasome activity in BPH-1 cell line

**Figure 2:**
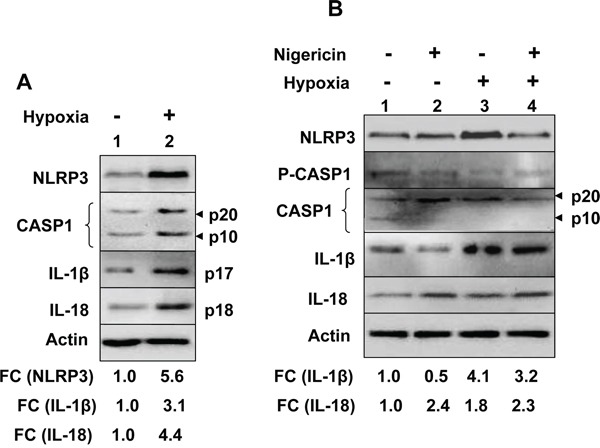
Hypoxia potentiated activation of the NLRP3 inflammasome activity in human benign prostate hyperplasia cell line BPH-1 **A.** Sub-confluent cultures of BPH-1 cell line were either incubated at normoxic (O_2_ levels 20.9%) or hypoxic (O_2_ levels 1.0%) chamber for 14 h. After the incubation, total cell lysates containing equal amounts of proteins were subjected to immunoblotting using antibodies specific to the indicated proteins. An arrow head in the right of figure indicates the protein band of interest, which is followed by an estimated molecular weight of the protein band. A consistent increase or decrease in levels of a protein in hypoxia-treated cells is marked by an upward arrow. Further, fold changes (FC) in the levels of the indicated proteins in hypoxia-treated PrECs were calculated as described in Methods. This ratio between the protein band signal and the actin protein band signal in control cells was indicated as 1. **B.** Cultures of BPH-1 cells as in the panel A, were either incubated under normoxic (lanes 1 and 2) or hypoxic (lanes 3 and 4) conditions for 14 h. After the incubation, cells were either treated with vehicle (lanes 1 and 3) or nigericin (10 μg/ml; lanes 2 and 4) for 45 min. Total cell lysates were prepared from control and treated cells and lysates containing equal amounts of proteins were analyzed by immunoblotting using antibodies specific to the indicated proteins. Fold changes (FC) in the levels of the indicated proteins in hypoxia-treated PrECs were calculated as described in Methods. This ratio between the protein band signal and the actin protein band signal in control cells was indicated as 1.

We also investigated hypoxia-induced induction of NLRP3 receptor and activation of the NLRP3 inflammasome in human benign prostate hyperplasia (BPH) cell line BPH-1, which exhibit a constitutively active NF-κB [35]. As shown in Figure 2A, hypoxia treatment of cells increased levels of NLRP3 receptor (5.6-fold), cellular levels of cleaved Caspase 1 (p20 and p10), cleaved IL-1β (p17) about 3-fold, and cleaved IL-18 (p18) about 4-fold. Further, treatment of normoxic and hypoxic BPH-1 cells with nigericin resulted in reduced cellular levels of procaspase-1 (p45), activated caspase-1 (p10; compare lane 4 with lane 2), and increased cellular levels of cleaved IL-1β (p17) and IL-18 (p18) (Figure 2B). Together, these observations suggested that hypoxia treatment of BPH-1 cells primed them as well as activated the NLRP3 inflammasome activity.

### Hypoxia primes and activates the NLRP3 inflammasome activity in a prostate cancer cell line

**Figure 3:**
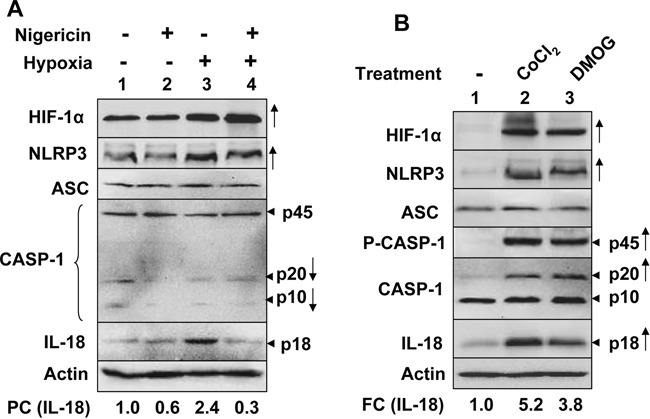
Hypoxia stimulated spontaneous and nigericin-induced NLRP3 inflammasome activity in PC-3 human prostate cancer cell line **A.** PC-3 cells as in the panel A, were either incubated under normoxic (lanes 1 and 2) or hypoxic (lanes 3 and 4) conditions for 18 h. After the incubation, cells were either treated with equal volume of ethanol (vehicle; lanes 1 and 3) or nigericin (10 μg/ml; lanes 2 and 4) for 45 min. Total cell lysates were prepared from control and treated cells and lysates containing equal amounts of proteins were analyzed by immunoblotting using antibodies specific to the indicated proteins. An arrow head in the right of figure indicates the protein band of interest, which is followed by an estimated molecular weight of the protein in kDa. An appreciable increase or decrease in levels of a protein in hypoxia-treated PC-3 cells (in at least two or more experiments) is marked by an upward or downward arrow, respectively, in the right side of the Figure A fold changes (FC) in the levels of cellular IL-18 in hypoxia-treated PC-3 cells was calculated as described in Methods. This ratio between the protein band signal and the actin protein band signal in control cells was indicated as 1. **B.** Sub-confluent cultures of PC-3 cells were either left untreated (lane 1) or treated with CoCl_2_ (100 μM) or Dimethyloxallyl Glycine (DMOG; 500 μM) to chemically-induce hypoxia for 14 h. Total cell lysates from control and treated cells were prepared and lysates containing equal amounts of proteins were analyzed by immunoblotting using antibodies specific to the indicated proteins. An arrow head in the right of figure indicates the protein band of interest, which is followed by an estimated molecular weight of the protein in kDa. An appreciable increase in the levels of the indicated proteins in hypoxia-treated cells is marked by an upward arrow in the right side of the Figure A fold changes (FC) in the levels of cellular IL-18 in hypoxia-treated PC-3 cells was calculated as described in Methods. This ratio between the protein band signal and the actin protein band signal in control cells was indicated as 1.

We also investigated whether hypoxia could prime and activate the inflammasome activity in PC-3 cells, which exhibit a constitutively active NF-κB activity [36]. As shown in Figure 3A, consistent with our above observations (Figure 1), treatment of PC-3 cells with hypoxia appreciable increased levels of NLRP3 receptor (Figure 3A, compare lane 3 with 1). However, the treatment reduced the cellular levels of the pro-caspase 1 (p45) and cleaved caspase 1 (p20), suggesting a spontaneous activation of caspase-1 and an inflammasome activity. Further, treatment of hypoxic PC-3 cells with nigericin appreciably decreased the cellular levels of IL-18 (p18) as compared with normoxic cells that were treated with nigericin (compare lane 4 with 2), thus suggesting that hypoxia treatment potentiated nigericin-induced activation of the NLRP3 inflammasome activity in PC-3 cells.

Because treatment of cells with hypoxia mimetic, such as CoCl_2_ [37] or dimethyl-oxaloylglycine (DMOG) [38] induces hypoxia in cells, we treated PC-3 cells with either CoCl_2_ or DMOG to further investigate whether chemically-induced hypoxia could spontaneously activate the inflammasome activity. As shown in Figure 3B, the treatment of cells with CoCl_2_ or DMOG increased HIF-1α protein levels appreciably. Further, the treatment increased levels of the NLRP3 receptor (compare lane 2 or 3 with 1). Notably, the treatment also activated caspase 1 activity, as determined by increases in cellular levels of cleaved caspase 1 (p20), and increased cellular levels of IL-18 (compare lane 2 or 3 with 1). Together, these observations indicated that an induction of hypoxia in PC-3 cancer cells primed cells and spontaneously activated NLRP3 inflammasome activity.

### Hypoxia activates inflammasomes activity in THP-1 myeloid cell line

**Figure 4:**
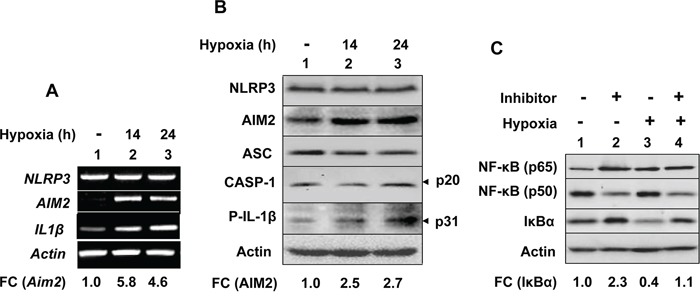
Hypoxia in human THP-1 monocytic cell line differentially regulated the expression of the NLRP3 and AIM2 receptors, and inflammasomes proteins through activation of the NF-κB activity **A.** THP-1 cell cultures in suspension were either incubated under normoxic (lane 1) or hypoxic condition for 14 (lane 2) or 24 h (lane 3). Total RNA was prepared and levels of the indicated mRNAs were analyzed by RT-PCR. FC indicates the fold-change in the levels of AIM2 mRNA in hypoxic as compared with normoxic THP-1 cells. **B.** THP-1 cells as in the panel (A) were either subjected to normoxic (lane 1) or hypoxic condition (lanes 2 and 3) for the indicated times. Total cell extracts were analyzed by immunoblotting for the indicated proteins. An arrow head in the right of figure indicates the protein band of interest, which is followed by an estimated molecular weight of the protein in kDa. A fold changes (FC) in the levels of cellular AIM2 in hypoxia-treated THP-1 cells was calculated as described in Methods. This ratio between the protein band signal and the actin protein band signal in control cells was indicated as 1. **C.** THP-1 cells in suspension were either treated with DMSO (vehicle; lanes 2 and 4) or Bay11-782 (12 μM) and cells were either incubated at normoxic (lanes 1 and 2) or hypoxic chamber for 14 h. Total cell lysates containing equal amounts of proteins were analyzed by immunoblotting for the levels of the indicated proteins. FC indicates the fold-change in levels of the IκBα protein.

HIF-1α is essential for myeloid cell (including macrophages)-mediated inflammation in tumors [39]. Therefore, we chose THP-1 cells, human monocytic cell line and a well-characterized cell model system to study inflammasomes activation [23]. Because PMA-treatment of THP-1 monocytic cells induces cell differentiation and increases the levels of HIF-1α and stimulates the expression of HIF-1α target genes [40], we used undifferentiated THP-1 monocytic cells to investigate whether hypoxia could “prime” activation of the NLRP3 or AIM2 inflammasome. As shown in Figure 4A, the treatment of cells with hypoxia (1% O_2_) for 14 or 24 h appreciably increased steady-state levels of AIM2, but not NLRP3, mRNA (compare lane 2 or 3 with lane 1). Further, the treatment for 14 h also increased levels pro-IL-1β mRNA (compare lane 2 or 3 with 1). Consistent with these observations, we also noted increases in levels of AIM2 receptor and pro-IL-1β proteins after hypoxia treatment (Figure 4B). These observations indicated that hypoxia in undifferentiated THP-1 cells “primed” cells for activation of the AIM2 inflammasome.

Hypoxia in myeloid cells activates the transcriptional activity of the NF-κB [14, 15]. Therefore, to investigate whether hypoxia “primed” activation of the AIM2 inflammasome activity in THP-1 cells through stimulation of the NF-κB activity, we pre-treated cells with BAY 11-7082, an inhibitor of NF-κB activation [41], and exposed cells to normoxia or hypoxia as described above. As shown in Figure 4C, pretreatment of normoxic cells with the inhibitor decreased constitutive levels of NF-κB (p50) and increased levels of IκBα inhibitor in cells (compare lane 2 with 1). Notably, hypoxia treatment of cells for 14 h increased levels of NF-κB (p65) and reduced levels of IκBα inhibitor (compare lane 3 with 1) and pretreatment of hypoxic cells with the inhibitor increased levels of IκBα inhibitor (compare lane 4 with 3). These observations indicated that hypoxia in THP-1 cells activated the NF-κB transcriptional activity.

**Figure 5:**
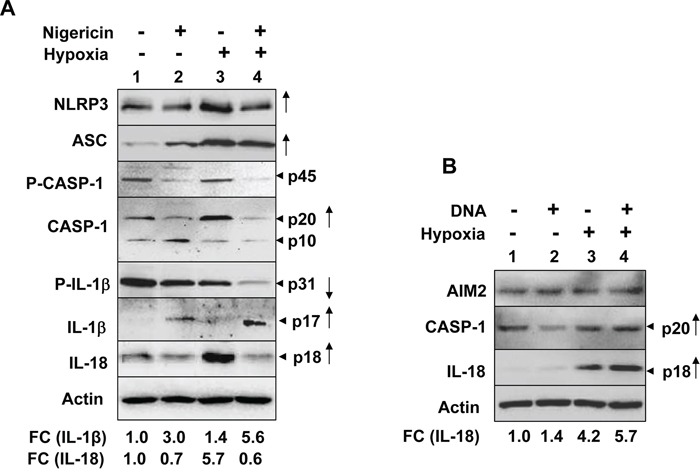
Hypoxia in PMA-differentiated THP-1 cells stimulated the spontaneous and ligand-induced activation of the NLRP3 and AIM2 inflammasome **A.** Suspension cultures of THP-1 cells were treated with 100 nM PMA for two days to induce differentiation. The differentiated cells were subjected to normoxia (lanes 1 and 2) or hypoxia (lanes 3 and 4) for 14 h. Following the treatment, cells were either treated with ethanol (vehicle; lanes 1 and 2) or nigericin (10 μg/ml; lanes 2 and 4) for 45 min. Total cell lysates were subjected to immunoblotting using antibodies specific to the indicated proteins. FC indicates the fold-change in the cellular levels of IL-1β (p17) and IL-18 (p18). An arrow head in the right of figure indicates the protein band of interest, which is followed by an estimated molecular weight of the protein band in kDa. An appreciable increase or decrease in levels of a protein in cell lysates in two or more experiments is marked by an upward or downward arrow, respectively, in the right side of the Figure **B.** Differentiated THP-1 cells as described in the panel (A) were subjected to normoxia (lanes 1 and 2) or hypoxia (lanes 3 and 4) for 14 h. Control and hypoxia-treated cells were either treated with transfection reagent LyoVec alone (lanes 1 and 2) or poly [dA:dT] LyoVec (25 μg/ml; lanes 2 and 4) for 4 h. Total cell lysates from control and treated cells were subjected to immunoblotting for the indicated proteins. FC indicates the fold-change in the cellular levels of IL-18 (p18). An arrow head in the right of figure indicates the protein band of interest, which is followed by an estimated molecular weight of the protein band in kDa. An appreciable increase in the levels of IL-18 in cell lysates (in 2 independent experiments) is marked by an upward arrow in the right side of the Figure.

To investigate whether hypoxia could potentiate activation of the NLRP3 or AIM2 inflammasome activity in differentiated THP-1 cells, we treated PMA-differentiated and LPS-primed THP-1 cells with either nigericin (an activator of NLRP3 inflammsome) or synthetic DNA (an activator of AIM2 inflammasome). As shown in Figure 5A, hypoxia treatment in the differentiated and “primed” THP-1 cells further increased the levels of NLRP3 receptor and ASC proteins. Further, the treatment increased basal as well as nigericin-induced activation of the NLRP3 inflammasome activity as determined by increases in cellular levels of cleaved caspase-1 (p20; compare lane 3 with 1), decrease in cellular levels of caspase-1 (p10; compare lane 3 with 1), decreases in cellular levels of pro-IL-1β (p31; compare lane 3 with 1), increases in cellular levels of IL-1β (p17; compare lane 4 with 2) and IL-18 (p18; compare lane 3 with lane 1 or lane 4 with lane 2). Similarly, hypoxia increased cellular levels of activated caspase-1 (p20; compare lane 4 with 2) and IL-18 (compare lane 4 with lane 2) in response to treatment with synthetic DNA (Figure 5B). Together, these observations revealed that hypoxia in differentiated THP-1 cells differentially potentiated activation of the NLRP3 and AIM2 inflammasome.

## DISCUSSION

Hypoxia in solid tumors is often associated with chronic inflammation and a poor outcome for cancer patients [5, 6, 8]. Although studies have indicated that infiltration of tumors by immune cells, including macrophages (referred as tumor-associated macrophages or TAMs), is associated with chronic inflammation [8], it remains unclear how cyclic or chronic hypoxia in normal epithelial and cancer cells contributes to chronic inflammation. Therefore, our observations that hypoxia in human normal PrECs (Figure 1C), immortalized BPH-1 cell line (Figure 2), and PC-3 prostate cancer cell line (Figure 3B) increased levels of NLRP3 receptor are novel. Further, treatment of PC-3 cells with hypoxia mimetic CoCl_2_ also appreciably increased levels of the NLRP3 receptor (Figure 3B). Notably, hypoxia also increased steady-state levels of IL-1β mRNA and protein in PrECs (Figure 1) and THP-1 cells (Figure 4). Of note, hypoxia increased the basal activation of caspase 1 in human normal PrECs (Figure 1C), BPH-1 cells line (Figure 2A), prostate cancer PC-3 (Figure 2A) and THP-1 myeloid cell lines (Figure 5A). These observations indicated that hypoxia treatment of these cells “primed” them for the activation of the NLRP3 inflammasome. Further, hypoxia also potentiated activation of NLRP3 and AIM2 inflammasomes, thus in part provided the “second” signal. Notably, our previous study [29] indicated that activation of the AIM2 inflammasome activity in human PrECs and PC-3 cells resulted in proteolytic cleavage of pro-IL-18 and its release from cells, our current observations do not rule out the possibility that the mechanisms other than the activation of an inflammasome by hypoxia in cells also contributed to the generation of mature IL-1β and IL-18.

BPH-1 and PC-3 prostate lines exhibit constitutively active NF-κB activity [18–20]. Because TLR-signaling mediated activation of the NF-κB transcriptional activity in myeloid cells “primes” cells (or provides the “first signal”) through the up-regulation of NLRP3 and pro-IL-1β protein expression [25, 27], the constitutive activation of the NF-κB activity in prostate cancer cells (such as PC-3 cell line) is predicted to “prime” cells for the activation of the NLRP3 inflammasome. Accordingly, PC-3 cells expressed higher levels of pro-inflammatory cytokines compared to LNCaP cells that exhibit reduced NF-κ B activity [18]. Therefore, our observations that hypoxia in human normal PrECs, BPH-1, and PC-3 prostate cell lines increased the spontaneous as well as the ligand-induced activation of the NLRP3 inflammasome are consistent with the previous observations.

Hypoxia in cells increases levels of reactive oxygen species (ROS) through mitochondrial complex III and increased levels of ROS stabilize HIF-1α [42]. Because increased levels of ROS can also provide a “second” signal for the activation of NLRP3 inflammasome [27, 28], it is likely that treatment of PrECs (Figure 1B), BPH-1 (Figure 2), PC-3 (Figure 2B), and THP-1 (Figure 4A) cells increased the basal activity of the NLRP3 inflammasome in part through an increased ROS production. Further work is in progress to test this interesting possibility.

Factors that are associated with pathological stress such as acute and chronic inflammation, infectious microorganisms, and tumor hypoxia stabilize HIF-1α protein [3–8]. Accordingly, IL-1β-induced signaling in myeloid cells increases levels of HIF-1α and stimulates its transcriptional activity [11, 13]. Notably, IL-1β increases the levels of HIF-1α through activation of transcriptional activity of the NF-κB [11]. The observation that HIF-1α can be activated in response to inflammatory cytokines may suggest a role for a feedforward loop between prostatic inflammation and HIF-1α in human prostate cancers. Therefore, our observations that hypoxia “primed” cells and potentiated activation of the NLRP3 and AIM2 inflammasomes in myeloid and non-myeloid cells are of significance to understand the role of hypoxia in the activation of inflammasomes and the development of PCI.

CoCl_2_ treatment-induced hypoxic condition in mixed glial cells, but not in bone marrow-derived macrophages, considerably inhibited NLRP3-dependent caspase-1 activation [43]. Similarly, CoCl_2_ treatment of isolated brain microglial cells also inhibited NLRP3 inflammasome activity. However, the treatment did not affect poly [dA: dT]-triggered AIM2 inflammasome activity [43]. These observations indicated that hypoxia-mediated activation of inflammasomes depends upon the cell type and the type of inflammasome. Consistent with the above report, our observations also revealed that hypoxia induced by reduced levels of oxygen (1% O_2_) in epithelial and myeloid cell lines differentially activated the NLRP3 and AIM2 inflammasomes activity.

Hypoxia in prostatic tumors is associated with tumor promotion, malignant progression, and resistance to cancer therapy [8, 19–21]. Further, hypoxia through activation of HIF promotes epithelial-to-mesenchymal transition (EMT), maintenance of cancer stem cell functions, and maintains the feedforward loop between hypoxia and inflammation [19–21]. Therefore, our observations that hypoxia treatment of human normal PrECs and myeloid cells constitutively activated an inflammasome activity and potentiated the ligand-induced activation of the NLRP3 and AIM2 inflammasome are significant. Further, these observations make it likely that activation of inflammasomes in hypoxic microenvironment of prostatic tumors through increased production of IL-1β induces stabilization of HIF-1α in tumor cells and promotes expression of hypoxia-inducible genes, thus activating a feedforward loop between prostatic hypoxia and prostatic inflammation. In summary, our observations will serve as basis to further investigate the role of tumor hypoxia in the activation of inflammasomes in prostate epithelial and cancer cells and their potential role in the development of chronic inflammation-associated prostatic diseases such as BPH and prostate cancer.

## MATERIALS AND METHODS

### Reagents

EDTA-free protease inhibitor cocktail was purchased from Roche Applied Science (Indianapolis, IN). Other reagents such as recombinant human IFN-γ (Minneapolis, MN), lipopolysaccharide (LPS) and phorbol 12-myristate 13-acetate (PMA) from Sigma-Aldrich (St. Louis, MO), synthetic double-stranded DNA [Poly(dA:dT)] in complex with transfection reagent (LyoVec) and nigericin from InvivoGen (San Diego, CA), and Bay11-782 were purchased from Sigma-Aldrich.

### Cell lines and treatments

Human normal prostate epithelial cells (PrECs; at passage 2) were purchased from Lonza (USA) and were maintained in culture as suggested by the supplier in the presence of medium supplements that were provided by the supplier along with the basal medium. BPH-1 cell line was provided by Dr. Simon Hayward (Vanderbilt University Medical Center, Nashville, TN). PC-3 prostate cancer cell line was originally purchased from ATCC.

Human monocytic cell line, THP-1, was originally purchased from the American Type Culture Collection (ATCC; Manassas, VA). These cells were maintained in suspension culture as suggested by ATCC. When indicated, for differentiation of cells, cell cultures were treated with 100 nM PMA for two days and the differentiated cells were “primed” with LPS (100 ng/ml, Sigma) for 3-4 h to increase levels of pro-IL-1β [44]. The primed cells were either left untreated or treated with nigericin (10 μg/ml), an activator of the NLRP3 inflammasome [34], for up to 45 min as described [44]. When indicated, differentiated cells were also treated with [Poly(dA:dT)] LyoVec (25 μg/ml), an activator of AIM2 inflammasome [29, 44], for up to 4 h. Total cells lysates were prepared and analyzed by immunoblotting for the cellular levels of activated caspase-1 (p20 and p10) and the mature IL-1β (p17) as described [44]. When indicated, cell culture supernatants were also collected and total proteins in the culture medium were precipitated. The precipitated proteins were boiled with sample buffer and samples containing approximately equal amounts of proteins were analyzed by immunoblotting for the secreted levels of IL-1β (p17) and IL-18 (p18).

### Hypoxia treatment

For hypoxia treatment of cells, sub-confluent cultures of cells were incubated at 1% O_2_ in a custom-designed, clear plastic hypoxia chamber (Biospherix, Lacona, NY) with a humidified environment at 37°C, 5% CO_2_, and continuous monitoring of oxygen levels by E-702 oxygen sensor (Biospherix). The ambient oxygen levels in the hypoxia chamber were decreased using a blended air/N_2_ gas mixture and the levels were adjusted continuously with a oxygen gas controller (Proox model-360; Bioshoerix) throughout during the incubation of cells. This set up required >6 h incubation time to achieve desired (1%) O_2_ levels. Because hypoxia chamber required a longer time to achieve desired levels of hypoxia, we also used hypoxia mimetic such as Cobalt Chloride (CoCl_2_; Sigma-Aldrich, St. Louis, MO) at a concentration of 100 μM or Dimethyloxallyl Glycine (DMOG; Sigma-Aldrich) at a concentration of 500 μM to chemically-induce hypoxia.

### Antibodies

Following specific antibodies were purchased to detect proteins in immunoblotting: ASC (sc-22514), IL-1β (sc-7884), and IL-18 (sc-7954) from Santa Cruz Biotech (Santa Cruz, CA); NLRP3 (HPA012878) from Sigma-Aldrich; Caspase-1 (AHZ0082) from Invitrogen (Grand Island, NY); β-actin (cat # 4967), IκBα (# 9247); and histone 3 (# 9715) from Cell Signaling Technology (Danvers, MA). Rabbit polyclonal antibodies to specifically detect two human hAIM2 isoforms that were raised by us have been described [29]. Horseradish peroxidase (HRP) conjugated secondary anti-mouse (NXA-931) and anti-rabbit (NA-934) antibodies were from GE Healthcare Biosciences (Piscataway, NJ).

### Immunoblotting

Cells pellets were suspended in desired volume of the radio-immunoprecipitation assay (RIPA) buffer (50 mM Tris-Cl (pH 7.4), 150 mM NaCl, 1% Nonidet P-40, 0.5% sodium deoxycholate, 0.1% sodium dodecyl sulfate) that was supplemented with complete mini EDTA-free protease inhibitor cocktail and phosphatase inhibitors (Cell Signaling, Danvers, MA). The lysates were incubated on ice for 30 min and briefly sonicated to release proteins from chromatin. Lysates were cleared by centrifugation at 12,000 rpm for 5 minutes at 4°C. Cell lysates containing approximately equal amounts of proteins (∼25-50 μg) were separated by SDS-PAGE, proteins were transferred to a polyvinylidene difluoride (PVDF; from Millipore) membrane, and immunoblotted as described [29]. When indicated, actin protein was used as an internal control (because levels of actin protein did not change after hypoxia treatment of cell types that we used). To calculate a fold change (FC) in levels of a protein of interest following hypoxia treatment, enhanced chemiluminescence (ECL) signals of actin protein (an internal control) and the protein of interest were measured by the Molecular Imager Gel Doc XR^+^ System (Bio-Rad, Hercules, CA) with the Image Lab Software. To estimate a relative FC in levels of a protein of interest after hypoxia treatment, the protein band signal for the protein of interest was divided by the actin protein band signal (which was normalized based on equal protein amounts per lane). This ratio between the protein band signal and the actin protein band signal in control cells was indicated as 1.

### Inflammasome assays

Activation of constitutive or induced activity of the inflammasome in myeloid and non myeloid cells was assessed using one or more of the following criteria [23]: (i) a decrease in the cellular levels of pro-caspase-1 (p45) due to its cleavage into the p20 and p10 forms; (ii) increases in cellular levels of caspase 1 p20 and/or p10; (iii) decreases in the cellular levels of pro-IL-1β (p31); and (iv) increases in the cellular levels of the mature IL-1β (p17); and (iv) increases in cellular levels of mature IL-18 (p18).

### RNA isolation and PCR

Cells pellets after centrifugations were suspended into Trizol reagent (Invitrogen) to isolate total RNA as described by the supplier. cDNA synthesis, semi-quantitative RT-PCR was performed to determine relative levels of mRNAs as described [29]. The following primers were used for semi-quantitative RT-PCR: human AIM2 (forward: 5′-atgtg aagccgtccaga-3′; backward: 5′-catcatttctga tggctgca-3′), human NLRP3 (forward: 5′-AGCC ACGCTAATGATCGACT-3′; backward: 5′-CAGGCTCAGAATGCTCATCA), human ASC (forward: 5′-GCCTGCA CTTTATA GACCAGC-3′; backward: 5′- GCTTCCGCATCTT GCTTGG-3′), human CASP-1 (forward: 5′-TCCAATAATGGA CAAGTC AAGCC-3′; backward: 5′-GCTGTACCCCAG ATTTTG TAGCA-3′), human IL-1β (forward: 5′-CTCGC CAGT GAAATGATG GCT-3′; backward: 5′-GTCGGAGATTC GTAGCTGGAT-3′) and actin (forward: 5′-GCTCGTCGT CGACA ACGGCTC-3′; backward: 5′-CATGATCTG GGTCA TCTTCTC-3′). The PCR cycling program consisted of denaturing at 95°C for 10 min and 40 cycles at 95°C for 15 seconds, and annealing and elongation at 60°C for 1 min. The signal in samples was normalized using the housekeeping genes as described [29]. Levels of actin mRNA were used as an internal control. To estimate a fold change (FC) in levels of an mRNA following a treatment, the intensity of the actin DNA band (an internal control) on the agarose gel and the DNA band of a gene of interest were measured by the Molecular Imager Gel Doc XR^+^ System (Bio-Rad, Hercules, CA) with Image Lab Software. Next, the ratio was calculated using the DNA band intensity value for the gene of interest and actin DNA band. This ratio in control cells was indicated as 1 and the FC for hypoxia treated samples was calculated by calculating the ratio between the value from treated samples (calculated as in the case of control sample) and the control value 1.

### Transfection

Cultures of PrECs in 60 mm plates at ∼50% confluence were either left untreated (control) or were “primed” with recombinant IFN-γ and TNF-α as noted above. Control or “primed” cells were either incubated with LyoVec (control) or poly(dA:dT)/LyoVec (5 μg/ml) for the indicated time. At the end of incubations, cells were harvested to prepare total cell lysates.

### Statistical methods

When indicated, experiments involving immunoblotting and semi-quantitative RT-PCR techniques were repeated at least 2-3 times and a representative result is shown. Fold-changes in the levels of certain proteins and mRNAs are indicated based on a representative experiment (out of 2-3 repeats).
